# Interaction Between Cadmium Stress and Sulphur Nutrition Level on Macronutrient Status of *Sinapis alba* L.

**DOI:** 10.1007/s11270-016-3059-9

**Published:** 2016-09-01

**Authors:** Renata Matraszek, Barbara Hawrylak-Nowak, Stanisław Chwil, Mirosława Chwil

**Affiliations:** 1 Department of Plant Physiology, Faculty of Horticulture and Landscape Architecture, University of Life Sciences in Lublin, Akademicka 15, 20-950 Lublin, Poland; 2 Department of Chemistry, Faculty of Agrobioengineering, University of Life Sciences in Lublin, Akademicka 15, 20-950 Lublin, Poland; 3 Department of Botany, Faculty of Horticulture and Landscape Architecture, University of Life Sciences in Lublin, Akademicka 15, 20-950 Lublin, Poland

**Keywords:** Cadmium, Macronutrient content and bioaccumulation, Sulphur nutrition level, White mustard

## Abstract

This study evaluated the possibility of improving the macronutrient status of Cd-stressed white mustard ‘Rota’ using intensive S nutrition. Three S-SO_4_ (2, 6, 9 mM S) and four CdCl_2_ doses (0, 0.0002, 0.02, 0.04 mM Cd) in the Hoagland’s nutrient solution were conducted for 14 days. High S supply (6 or 9 mM) appears, to some extent, to affect positively the macronutrient status of Cd-stressed mustard. It increased roots and shoots contents of K and S, without significant changes in P content. Simultaneously, Mg content in shoots and roots remained stable, but Mg bioaccumulation was elevated. Shoot Ca content at the lowest and medium Cd dose decreased, whilst was unaffected at the highest Cd treatment. Intensive S nutrition of Cd-stressed mustard increased root N content and accumulation at the highest Cd concentration, but the N content dropped in above-ground parts. The bioaccumulation of remained macronutrients in general was substantially elevated together with enhanced Cd accumulation. Thus, the intensive S nutrition can enhance mustard tolerance to Cd stress by improvement macronutrients relations in plants, and S supplementation may be recommended for mustard cultivation on the Cd-contaminated areas.

## Introduction

White mustard, also known as yellow mustard (*Sinapis alba* L. syn. *Brassica hirta* Moench, *B. alba* L.), is native to southern Europe, and the cultivation of this oilseed species has spread to a majority of temperature regions of the world, mostly in Canada and Nepal, which are the global leaders of white mustard. Ukraine, Russia, Czech Republic, Italy, the UK and the Netherlands are mentioned as the European leaders in the production of this species (Prakash et al. 2011; Jankowski et al. 2015). Production assessment of this important agricultural oil seed crop species in Poland is not easy on account of the prevailing cultivation of this species as a catch crop. It is estimated that in this country oilseed crops, i.e. white mustard together with sunflower and poppy, excluding barring rape, cover an area 24,000 ha. It is presumed that the importance and hence the cultivation and production of this multifunctional species in the nearest years will increase markedly (Dukarska et al. 2011). White mustard seeds are used in the food, pharmaceutical and cosmetics industries. First of all, they serve to obtain table mustard, oil and various kinds of spices. Additionally, fresh mustard greens are often eaten raw in the form of salad or juice (Manohar et al. 2009; Dukarska et al. 2011). Mustards are widely used in agriculture and horticulture as green manure, fodder crop as well as winter or rotational cover crops in production of various species. Similar to the other members of the Brassicaceae family, since they provide allelopathic compounds in a process termed ‘biofumigation’, mustards may control weeds as well as a range of soil-borne pests, pathogens and diseases. This process is related to release of volatile toxic isothiocyanate compounds (ITCs) through the degradation of secondary metabolites called glucosinolates (GLS) (Motisi et al. 2009).

It is widely known that members of the Brassicaceae family are characterized by high requirements for sulphur (S) due to oil production and GLS synthesis. Besides the high S level, a proper range of the N/S ratio from 4:1 to 8:1 is needed to obtain high yield and quality. Mustard requires constantly available S throughout the whole vegetation period with a higher requirement at the flowering stage, as S is a major constituent of seed protein. It is estimated that mustard seeds contain about 40 % S included in the stems (Barczak et al. 2011; Piri 2012; Singh et al. 2012a). An insufficient S level in the environment and hence reduced yield and quality is a globally widespread phenomenon. It is related to progressive reduction of emissions of S compounds to the natural environment, increasing use of S-free NPK fertilizers, as well as more and more intensive crop production. Furthermore, SO_4_^2−^ ions easily leach deeper into the soil profile and are relatively immobile in the soil-plant system, which contributes to the limited availability thereof to plants (Mašauskiene and Mašauskas 2012). S is needed for chlorophyll synthesis and is crucial to proper cell metabolic pathways such as the electron transport in iron-sulphur clusters, redox cycle, protein disulphide bridges and metabolism of secondary products (GLS and allyl Cys sulfoxides). First of all, S is a constituent of amino acids and hence proteins. Besides proteins, S is also an important constituent of lipids, polysaccharides, vitamins and cofactors (Bashir et al. 2015; Rathore et al. 2015). A suitable S level is important to plants not only for proper growth and development but also for enhanced tolerance to many stress factors (Nocito et al. 2006, 2007; Gill and Tuteja 2011). It is widely known that ligands containing SH-groups, i.e. tripeptide glutathione (GSH) or phytochelatins (PCs), form high-strength, durable complexes with heavy metals. In spite of extensive research, the role of S-rich secondary metabolites in detoxification of heavy metals is still discussed with unclear conclusions (Rivera-Becerril et al. 2005; Mazid et al. 2011; Postrigan et al. 2013).

Cadmium (Cd) is a strongly phytotoxic trace metal, which is very easily taken up by plants and incorporated to the food chain causing serious threats for all living organisms. The main anthropogenic sources of this metal are mining and refining of nonferrous metals, manufacture and application of phosphate fertilizers, fossil fuel combustion, as well as incineration and disposal of waste. Cd accumulation in plants alters mineral nutrient uptake and distribution, negatively affects water status, disturbs photosynthesis, interferes with the carbohydrate metabolism and indirectly induces oxidative stress. In the plant-soil relationship, Cd may affect various physiological and biochemical processes mainly by disturbing mineral nutrient balance and functions. Recognition of the interactions between Cd and mineral nutrients is a crucial step towards optimization of mineral nutrients for Cd toxicity alleviation. It is known that Cd interacts with the availability of essential elements and, on the other hand, some essential elements may play a protective role against Cd stress (Rivera-Becerril et al. 2005; Sarwar et al. 2010; Nazar et al. 2012; Przedpełska-Wąsowicz et al. 2012; Gharaibeh et al. 2016). Similar to the other members of the Brassicaceae family, the white mustard may tolerate excessive concentrations of heavy metals, including Cd. Gvozdenac et al. (2013) revealed that white mustard is not sensitive to Cd when present in water in amounts lower than maximal allowable amounts, i.e. in surface and underground waters of II and III class 0.0077–0.0102 μM and in irrigation water 0.1704 μM. They showed that germination of white mustard seeds was reduced in the presence of 0.017 μM Cd. In turn, root growth was inhibited at 3.41 μM Cd, whilst shoot length was stimulated at a concentration below 0.17 μM Cd.

The purpose of this study was to investigate the changes in macronutrient content of Cd-stressed white mustard (*Sinapis alba* L.) supplied with various S doses and thereby to evaluate the possibility of improving the macronutrient status of plants altered by Cd presence using intensive S nutrition. The results presented in this manuscript are only part of an extensive project (unpublished work) concerning the role of intensive S nutrition in Cd tolerance. We hope that our investigations may be helpful in further studies on developing strategies in alleviation of Cd phytotoxicity and they may find practical application, which is important for farmers.

## Materials and Methods

### Plant Material Characteristics and Growth Conditions

The experiment was conducted in the years 2011–2014 in the vegetation room of the Plant Physiology Department (University of Life Sciences in Lublin, Poland) on white mustard ‘Rota’. This cultivar was entered into the Polish National List in COBORU 24.02.2003—registration number R 1304 (Polish National List of Agricultural Plant Varieties 2014). It is characterized by high productivity, good health status and high tolerance to periodic water deficit. Plants of this cultivar are medium in height with low lodging resistance. With greater efficiency than that of other cultivars, it reduces by 27 % the population of sugar beet nematode (*Heterodera schachtii* Scm.). For this reason, it is a very valuable variety for post-cultivation of sugar beet, potatoes as well as other root crops and vegetables (Polish National List of Agricultural Plant Varieties 2014).

The white mustard seeds were germinated in quartz sand, and then the 1-week-old, best-developed seedlings were transferred to 1 dm^3^ glass jars (two plants per jar) filled with full-strength Hoagland’s No. 2 nutrient solution (Hoagland and Arnon 1950) modified in regard to S and Cd concentrations. Three sulphate (VI) sulphur doses (2 mM S—as standard concentration, 6 and 9 mM S—as high concentrations) and four levels of cadmium (II) chloride (0, 0.0002, 0.02 and 0.04 mM Cd) were used. The standard S dose was supplied as MgSO_4_, but in high-S treatments, this macronutrient was supplemented as MgSO_4_ (2 mM) and appropriate amounts of Na_2_SO_4_ (4 or 7 mM). In all experimental treatments, the level of sodium (Na) and chlorine (Cl) in the medium was equalized by adding appropriate amounts of NaCl or HCl; the pH of the nutrient solution was set at 5.8–6.0. Plants were grown in an air-conditioned phytotron room, with a 14-h photoperiod at Photosynthetic Photon Flux Density (PPFD) 400 ± 10 μmol m^2^ s^−1^, a day/night temperature of 25/20 °C, and 60–70 % relative air humidity. The nutrient solution was aerated for 15 min every 3 days and replenished with fresh nutrient solution, when the medium level was depleted to about 70 % of the initial level. After 2 weeks of growth under conditions of different Cd and S concentrations, plants were harvested. The roots from the Cd treatments were immediately washed in a 10 mM CaCl_2_ solution for 10 min and then twice in distilled water in order to remove the cell wall-bound Cd ions, whilst roots of plants grown without Cd were washed in distilled water. The dry mass (data in press), the macronutrient composition and Cd concentration in roots and shoots were evaluated.

### Macronutrient Content

The dry plant material (roots and shoots separately) was subjected to chemical analyses to determine the content of the macronutrients. Total nitrogen (N) was assayed with the classic Kjeldahl method; phosphorus (P) with the molybdenum vanadate colorimetric method; and potassium (K), calcium (Ca) and magnesium (Mg) with the atomic absorption spectrometry (AAS) technique after wet mineralization in the mixture of concentrated H_2_SO_4_ and 30 % H_2_O_2_. Total S was determined by modified nephelometric Butters-Chenery method. The Butters-Chanery method was a modified by using an aqueous solution of BaCl_2_ (instead of solid BaCl_2_) and a surfactant stabilizer of the suspension formed in the presence of HCl (Bielecki and Kulczycki 2012). The results concerning macronutrient content together with the productivity data (dry mass) were used to calculate the accumulation of macronutrients in roots and shoots (Melo et al. 2013).

### Cadmium Determination

Total Cd measurements were performed by an accredited laboratory of the Regional Chemical–Agricultural Station in Lublin. Dried shoot and root samples were subjected to nitric–perchloric acids mineralisation (HNO_3_–HClO_4_; 4:1; *v*/*v*) at 210 °C. The content of Cd was determined using the classic AAS technique (GBC Avanta, Sigma). In this work, the data concerning the Cd accumulation have been presented. Cd accumulation in shoots and roots was calculated based on the Cd content and dry biomass (Pereira et al. 2011).

### Statistical Analysis

The experimental design was randomized with 12 treatments and 20 replicates per treatment. Each experimental unit included 60 plants (ten jars in each treatment with two plant in each jar, and the experiment was repeated three times under the same conditions). Three measurements in each treatment were made per each independent repetition of the experiment over the time. A two-way ANOVA, with S and Cd concentrations in the nutrient solution as a differentiating factor, was used to compare the results, followed by the post hoc multiple comparisons of means using Tukey’s test. All calculations were performed using software package STATISTICA 9 and the differences were considered significant at *p* ≤ 0.05. The values in the same treatment and mean values among each treatment obtained from each of the three independent replicates of the experiment over the time did not differ significantly. Therefore, the data presented in the table and in the figures represent mean values with together with marked standard deviation (±SD).

## Results and Discussion

### Macronutrient Content and Accumulation

The lowest Cd concentration used in our studies (0.0002 mM) did not exceed the toxic values to phytoplankton and aquatic animals (0.0003–0.0007 mM), but exceeded twice the allowable level of this metal in drinking water. A concentration 0.0008 mM was recognized as a critical Cd limit for soil solution (De Vries et al. 2007). In terms of the Cd concentration in the soil solution, it is estimated that a non-polluted and polluted at a moderate level soil solution contain from 0.00004 to 0.00032 and from 0.00032 to 0.001 mM Cd, respectively (Hoseini and Zargari 2013).

Statistical analysis of main effects showed that the Cd presence in the nutrient solution (0.0002, 0.02 or 0.04 mM), irrespective of the S level, in general substantially raised the root and dropped the shoot N, Ca and S contents, including among other exceptions elevated N content under the lowest Cd concentration used (Table 1). The changes in N content were much more marked in roots than in shoots, whilst the opposite tendencies were shown for the Ca and S levels. As a rule, the Mg content decreased in roots, but did not change in shoots. Additionally, the P content in the biomass of Cd-stressed mustard remained unchanged except for the elevated root P content at 0.02 mM Cd. Simultaneously, the decrease in the root K content was accompanied by an increase (at 0.0002 mM Cd), a decrease (at 0.02 mM Cd), and no significant changes (at 0.04 mM Cd) in the K shoot level (Table 1). Based on the changes in the Ca and Mg contents, it may be assumed that the uptake of these nutrients is a non-specific process, which is in accordance with the findings of Jiang et al. (2004) for Cd-contaminated Indian mustard. Moreover, they recorded a significant increase in the root K and P contents and a decreased level of these nutrients in shoots. A trend similar to our findings concerning the N content in above-ground parts and an opposite tendency in roots were shown by Ciećko et al. (2004) for Cd-exposed maize, yellow lupine and lacy phacelia. Quite similar tendencies to that recorded for total S were shown by Sun et al. (2007) in oilseed rape. Reduced S and Ca contents in white mustard as a consequence of strong inhibition of their uptake under Cd exposure was also revealed by Metwally et al. (2005) for pea.

**Table 1:** The content of macronutrients (means ± SD, *n* = 9) in the organ biomass of white mustard ‘Rota’ exposed to different sulphur (S) and/or cadmium (Cd) concentrations in the nutrient solution

Concentration of the element in the nutrient solution (mM)			Content of the macronutrients in the biomass (g kg^−1^ DW)											
			N		P		K		Ca		Mg		S	
S	Cd		Roots	Shoots	Roots	Shoots	Roots	Shoots	Roots	Shoots	Roots	Shoots	Roots	Shoots
2	0.00		9.28 ± 0.19^i^	37.13 ± 1.22^ab^	5.94 ± 0.36^c^	0.87 ± 0.04^ab^	18.31 ± 1.05^de^	29.45 ± 1.53^a–c^	1.72 ± 0.05^fg^	10.56 ± 0.75^cd^	1.08 ± 0.10^ab^	2.78 ± 0.14^a^	12.34 ± 1.22^b^	17.35 ± 0.45^d–f^
6			11.07 ± 0.33^g^	29.35 ± 1.47^c–f^	5.32 ± 0.25^cd^	0.94 ± 0.10^ab^	20.17 ± 0.75^a–e^	21.78 ± 1.07^f–h^	1.67 ± 0.07^g^	12.89 ± 1.12^ab^	0.97 ± 0.05^a–c^	1.92 ± 0.20^c^	20.55 ± 1.36^a^	19.68 ± 0.53^b–d^
9			10.39 ± 0.2^gh^	31.46 ± 1.03^b–e^	6.36 ± 0.17^bc^	0.89 ± 0.07^ab^	19.57 ± 0.92^b–e^	22.51 ± 1.23^fg^	1.88 ± 0.04^d–f^	13.57 ± 0.58^a^	1.12 ± 0.07^a^	2.17 ± 0.09^bc^	21.10 ± 1.09^a^	19.97 ± 0.37^b–d^
2	0.0002		12.72 ± 0.13^ef^	42.27 ± 1.75^a^	5.34 ± 0.20^cd^	0.81 ± 0.09^ab^	17.87 ± 0.86^e^	25.37 ± 1.76^c–f^	1.85 ± 0.03^ef^	11.23 ± 1.04^bc^	1.12 ± 0.08^a^	2.57 ± 0.17^ab^	13.46 ± 1.17^b^	16.68 ± 0.46^ef^
6			13.67 ± 0.24^de^	32.78 ± 1.31^b–e^	4.78 ± 0.29^d^	0.98 ± 0.08^ab^	20.65 ± 0.49^a–d^	33.98 ± 1.23^a^	2.08 ± 0.05^bc^	7.69 ± 0.46^ef^	0.95 ± 0.04^a–c^	2.12 ± 0.13^bc^	20.28 ± 0.93^a^	16.88 ± 0.55^d–f^
9			11.56 ± 0.18^fg^	36.25 ± 1.14^a–c^	5.97 ± 0.24^a–c^	1.08 ± 0.11^a^	20.76 ± 0.37^a–c^	31.89 ± 1.04^ab^	1.94 ± 0.04^c–e^	8.47 ± 0.34^ef^	1.05 ± 0.07^ab^	2.27 ± 0.07^a–c^	22.99 ± 1.05^a^	18.90 ± 0.34^c–e^
2	0.02		16.54 ± 0.25^ab^	34.14 ± 1.27^b–d^	6.87 ± 0.12^ab^	0.85 ± 0.08^ab^	11.84 ± 0.67^f^	17.58 ± 0.74^h^	2.47 ± 0.06^a^	8.25 ± 0.61^ef^	0.87 ± 0.05^bc^	2.63 ± 0.05^ab^	13.68 ± 1.28^b^	15.99 ± 0.22^f^
6			15.48 ± 0.12^bc^	23.06 ± 1.55^f^	6.01 ± 0.19^a–c^	0.80 ± 0.06^ab^	18.82 ± 0.61^c–e^	22.78 ± 0.62^e–g^	1.56 ± 0.03^g^	5.89 ± 0.41^g^	0.76 ± 0.09^cd^	2.25 ± 0.11^a–c^	23.89 ± 0.82^a^	21.71 ± 0.29^a^
9			14.78 ± 0.10^cd^	25.87 ± 1.48^ef^	6.82 ± 0.26^ab^	0.88 ± 0.09^ab^	22.23 ± 0.77^a^	24.16 ± 1.14^d–f^	2.21 ± 0.07^b^	6.83 ± 0.88^fg^	0.79 ± 0.08^cd^	2.46 ± 0.06^a–c^	22.46 ± 1.33^a^	20.98 ± 0.70^a–c^
2	0.04		10.65 ± 0.21^g^	36.51 ± 1.37^a–d^	3.68 ± 0.14^e^	0.79 ± 0.05^ab^	12.42 ± 0.45^f^	18.18 ± 0.81^gh^	1.23 ± 0.04^h^	7.81 ± 0.93^ef^	0.63 ± 0.03^d^	2.81 ± 0.09^a^	20.68 ± 1.08^a^	11.35 ± 0.40^g^
6			14.46 ± 0.16^cd^	27.67 ± 1.84^d–f^	5.82 ± 0.31^b–d^	0.73 ± 0.08^b^	21.56 ± 0.53^ab^	28.42 ± 1.49^b–d^	1.89 ± 0.06^de^	8.46 ± 0.75^ef^	0.82 ± 0.09^cd^	2.39 ± 0.13^a–c^	22.29 ± 0.89^a^	15.74 ± 0.32^f^
9			16.89 ± 0.14^a^	30.42 ± 1.02^b–e^	7.02 ± 0.22^a^	0.89 ± 0.05^ab^	18.78 ± 0.57^c–e^	27.56 ± 1.36^b–e^	2.02 ± 0.04^cd^	9.38 ± 0.32^de^	0.83 ± 0.06^cd^	2.63 ± 0.15^ab^	23.79 ± 1.14^a^	22.99 ± 0.63^a^
Main effects														
S		2	12.30 ± 0.09^b^	37.51 ± 1.25^a^	5.46 ± 0.13^b^	0.83 ± 0.03	15.11 ± 0.25^b^	22.65 ± 0.91^b^	1.82 ± 0.03^b^	9.46 ± 0.25^a^	0.93 ± 0.05	2.70 ± 0.10^a^	15.04 ± 0.61^b^	15.34 ± 0.54^c^
		6	13.67 ± 0.13^a^	28.22 ± 1.08^b^	5.48 ± 0.11^b^	0.86 ± 0.04	20.30 ± 0.38^a^	26.74 ± 0.80^a^	1.80 ± 0.04^b^	8.73 ± 0.29^b^	0.88 ± 0.04	2.17 ± 0.05^b^	21.75 ± 0.78^a^	18.50 ± 0.36^b^
		9	13.41 ± 0.15^a^	31.00 ± 1.01^b^	6.30 ± 0.19^a^	0.93 ± 0.04	20.34 ± 0.33^a^	26.53 ± 0.77^a^	2.01 ± 0.04^a^	9.56 ± 0.20^a^	0.95 ± 0.05	2.38 ± 0.07^b^	22.59 ± 0.83^a^	20.71 ± 0.48^a^
Cd		0	10.25 ± 0.17^d^	32.65 ± 1.16^b^	5.87 ± 0.22^b^	0.90 ± 0.62	19.35 ± 0.44^a^	24.58 ± 0.04^b^	1.76 ± 0.04^c^	12.34 ± 0.43^a^	1.06 ± 0.04^a^	2.29 ± 0.14	18.00 ± 0.65^c^	19.00 ± 0.47^a^
		0.0002	12.65 ± 0.21^c^	37.10 ± 1.35^a^	5.36 ± 0.20^b^	0.96 ± 0.58	19.76 ± 0.40^a^	30.41 ± 0.06^a^	1.96 ± 0.03^b^	9.13 ± 0.37^b^	1.04 ± 0.05^a^	2.32 ± 0.10	18.91 ± 0.60^c^	17.49 ± 0.38^b^
		0.02	15.16 ± 0.27^a^	27.69 ± 1.04^c^	6.57 ± 0.27^a^	0.84 ± 0.71	17.63 ± 0.33^b^	21.51 ± 0.06^c^	2.08 ± 0.04^a^	6.99 ± 0.29^c^	0.81 ± 0.02^b^	2.45 ± 0.13	20.01 ± 0.87^b^	19.56 ± 0.51^a^
		0.04	14.00 ± 0.23^b^	22.53 ± 1.25^d^	5.51 ± 0.18^b^	0.80 ± 0.56	17.59 ± 0.31^b^	24.72 ± 0.03^b^	1.71 ± 0.04^c^	8.55 ± 0.33^b^	0.76 ± 0.03^b^	2.61 ± 0.11	22.25 ± 0.94^a^	16.69 ± 0.42^b^
Statistical significance														
S			*	*	*	NS	*	*	*	*	NS	*	*	*
Cd			*	*	*	NS	*	*	*	*	*	NS	*	*
S × Cd			*	*	*	*	*	*	*	*	*	*	*	*

Different letters within the same column denote significant differences between means of nine replications according to the Tukey’s multiple range test (*p* ≤ 0.05). Significant effects for the main factors and the interactions between them are denoted with asterisks (*)

*NS*, not significant

The mechanism of the Cd effect on nutrient uptake is not fully clear. It is postulated that the unfavourable changes in the essential element content in the Cd-stressed plants are related to the fact that Cd uptake occurs through transporters for essential elements (Jiang et al. 2004; Rivelli et al. 2014). Cd reduces the activity of H^+^ATPase—an ion transporter protein across the plasma membrane—and changes the electrochemical gradient of the membrane, which leads to lower absorption of some necessary nutrients (Astolfi et al. 2005). Furthermore, the inhibition of iron (Fe) chelation and loading to the xylem as well as competition for Ca transporters are proposed as the major mechanisms of Cd-induced disturbance in ion balance (Tran and Popova 2013). Nutrient disorders in Cd-stressed plants may also be related to the restricted water uptake and movement as well as inhibition of respiration and low status of energy needed for the active uptake and nutrient transport (Nazar et al. 2012; Tran and Popova 2013).

The S-SO_4_ level in the nutrient solution, besides Cd, was the second factor differentiating the conditions of the experiment, which was used in order to improve Cd-stressed mustard nutrient status. The S-SO_4_^2−^ concentration in a natural environment, i.e. unpolluted with heavy metals, oscillates between 0.16 and 7 mM, in arid regions between 3 and 16 mM, whilst in soil solutions with residues of sulphide ore mine between 13 and 110 mM (Nocito et al. 2006; Sun et al. 2007). The standard nutrient solution, for example Hoagland’s, contains 2 mM S in the form of S-SO_4_. Such a S level was used in our experiment as a standard level. It is believed that after cultivation of plant characterized by high requirements for S such as mustard, the concentration of this macronutrient may decrease even to the deficit level, which leads to reduction in S-SO_4_ uptake and changes in the proper S metabolism pathway and, consequently, a drop in thiol synthesis (Sun et al. 2007; Ernst et al. 2008). There is no doubt that upregulation of the S-assimilation pathway is considered a crucial step for tolerance and survival of Cd-stressed plants (Nocito et al. 2007; Gill et al. 2011; Nazar et al. 2012).

Two mechanisms: direct and indirect of Cd alleviation by nutrient supplementation are proposed. The first one is associated with the competition between essential elements and Cd for the same transporters, reducing the availability of Cd by facilitating precipitation and adsorption as well as sequestration of Cd in the cell compartments of the vegetative organs. The second one involves dilution of Cd ions due to enhanced production of biomass (Rahman et al. 2007; Tran and Popova 2013). Alleviation of Cd phytotoxicity by S supplementation occurs either through decreasing uptake or reducing Cd toxicity. In limiting Cd accumulation, the most important role is played by ATP-sulphydrylase (ATPS) and serine acetyl transferase (SAT). High ATPS activity expression is required for increased glutathione (GSH) content, which together with an elevated ascorbate (AsA) level is needed for proper functioning of the AsA/GSH cycle and, as a result, reduced Cd toxicity. GSH is involved in Cd homeostasis (via synthesis of PCs), antioxidative defence and signal transduction under Cd stress. The diverse functions of GSH are related to the cysteine sulphydryl group (-SH), enabling GSH to chelate metals and participate in red-ox cycling. Cd is detoxified in the cytosol by forming durable Cd-PCs complexes, which are compartmentalized into vacuoles (Nocito et al. 2007; Sarwar et al. 2010; Gill et al. 2011; Jozewczak et al. 2012). Similarly, our results (data in press) indicate the decrease in the GSH content accompanied by increased PC accumulation in high-S-supplemented (6 or 9 mM S) white mustard exposed to Cd.

Intensive S nutrition of white mustard grown in the medium without Cd resulted in an increase in the N and S contents in roots without changes in the P, K, Ca and Mg levels (Table 1). Under these conditions in shoots, a reduction in N, K and Mg contents was found together with an increase in Ca and no significant changes in the P and S levels (Table 1). The results of our studies support a rule that the content of essential elements in the biomass increases together with their increasing level in the nutrient solution (Jat and Mehra 2007; Jankowski et al. 2015). The increased total S content in the shoot and root biomass of intensively fertilized with S white mustard was recorded irrespective of the Cd concentration. Similarly, Vestena et al. (2007) revealed that the S content was always higher in highly S-supplemented water hyacinth and salvinia, irrespective of the presence of Cd. Moreover, our results revealed that intensive S nutrition of Cd-treated white mustard, irrespectively to the Cd concentration in the nutrient solution, markedly raised the root and dropped shoot N content, and the changes in the shoot N content were more marked than those in roots (Table 1). Simultaneously, in roots and shoots, an increase in the K and S contents was found, without changes in P content (except for the rise in the root P content at the highest S treatment). It is worth stressing that the increase in the K and S contents was more pronounced in roots than in shoots. Moreover, there was a significant decrease in shoot Mg content together with unchanged root Mg content. It was also shown that, regardless of the Cd concentration, the dose of 6 mM S did not change the content of Ca in roots and decreased its content in shoots, whilst the Ca content at 9 mM S was elevated or remained quite stable in roots and shoots, respectively (Table 1). Therefore, our study shows a synergistic effect between S and K and an antagonistic relationship between S and Mg as well as S and N in Cd-stressed white mustard. However, the tendencies in the changes in the root and shoot N content were opposite as the content of this macronutrient raised in under- and dropped in aboveground parts. In turn, the relationship between S and Ca in Cd-exposed mustard was not clear and depended on the S concentration. In the presence of 6 and 9 mM S, antagonistic and synergistic effects between these macronutrients, respectively, were revealed.

**Fig. 1:**
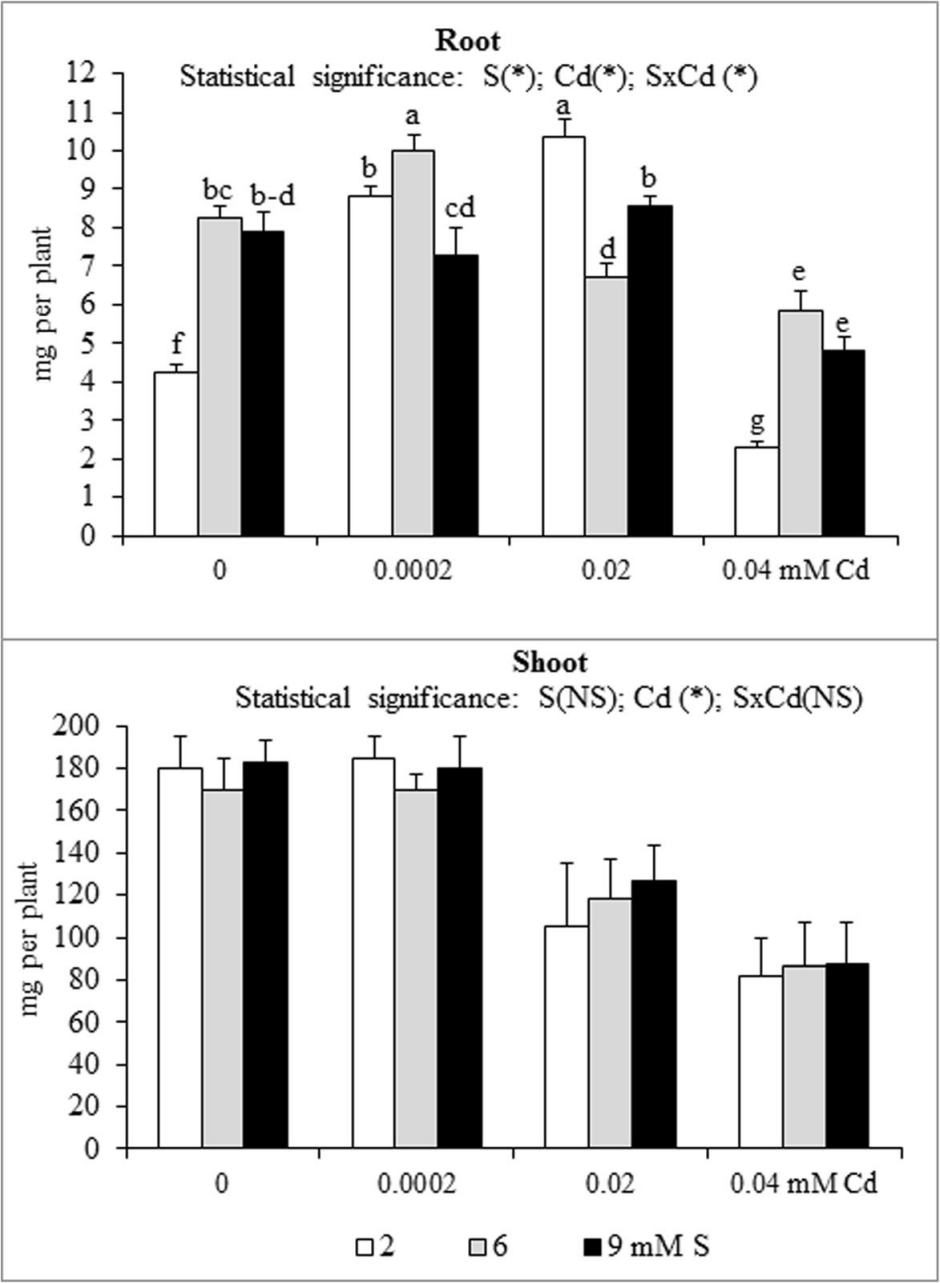
Nitrogen accumulation in the in the biomass of white mustard ‘Rota’ grown under different sulphur (S) and/or cadmium (Cd) concentrations in the nutrient solution. Mean values (*n* = 9) marked with the same letter are not different at *p* ≤ 0.05 based on the Tukey’s test. Significant effects for the main factors and the interactions between them are denoted with *asterisks. Vertical bars* represent the standard deviation of means

**Fig. 2:**
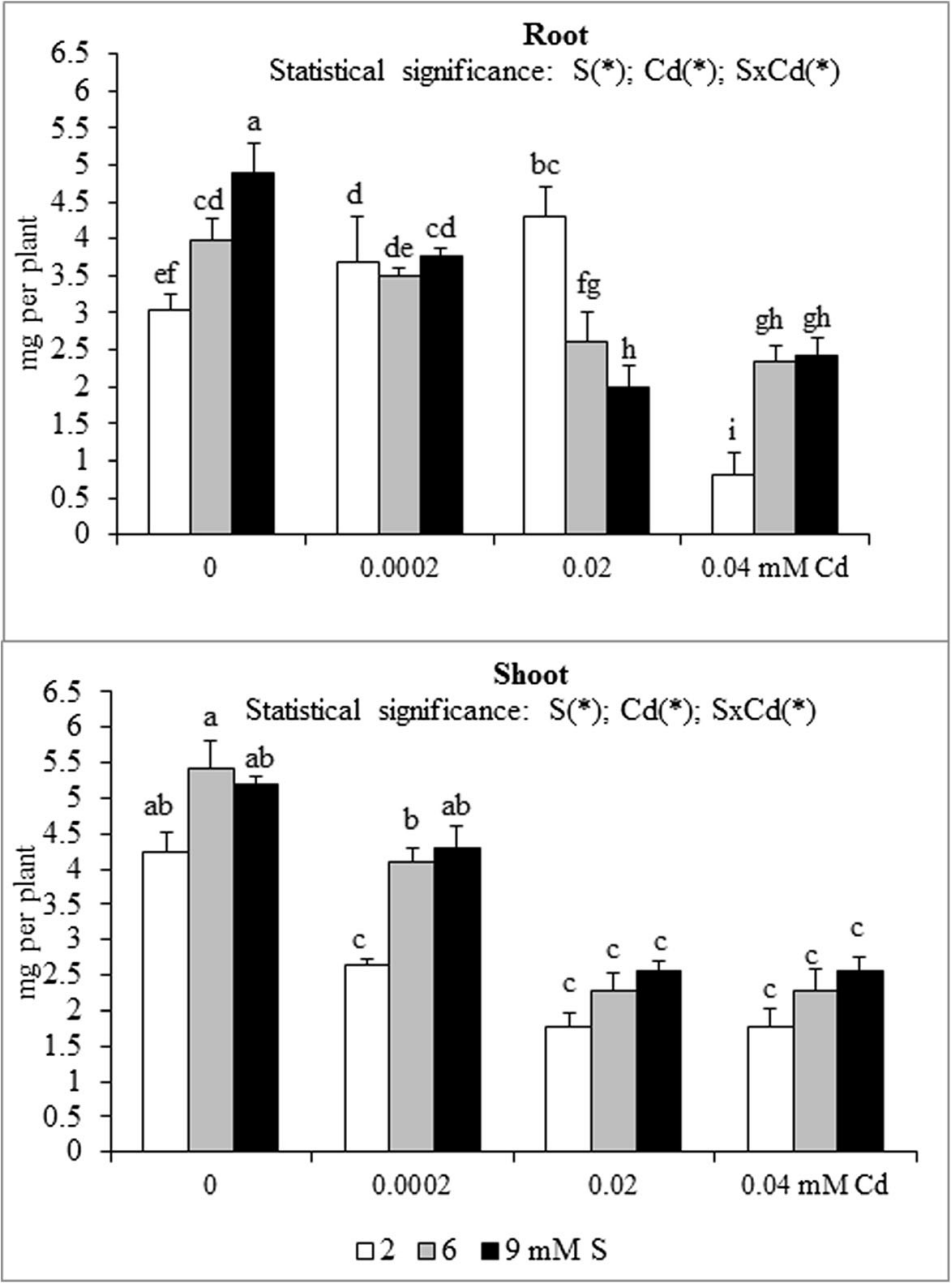
Phosphorus accumulation in the in the biomass of white mustard ‘Rota’ grown under different sulphur (S) and/or cadmium (Cd) concentrations in the nutrient solution. Note: Explanations are the same as in Fig. 1

**Fig. 3:**
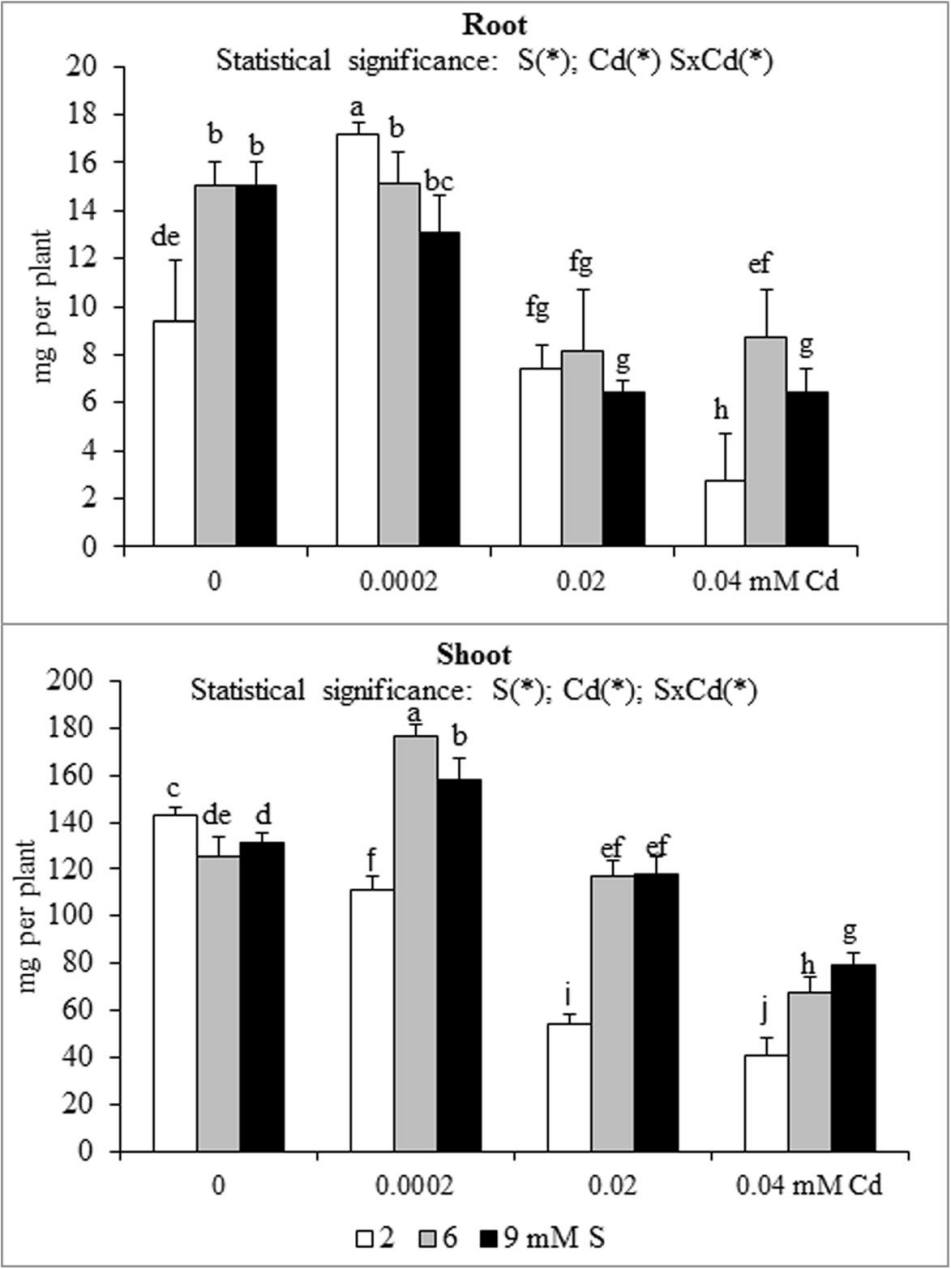
Potassium accumulation in the in the biomass of white mustard ‘Rota’ grown under different sulphur (S) and/or cadmium (Cd) concentrations in the nutrient solution. Note: Explanations are the same as in Fig. 1

**Fig. 4:**
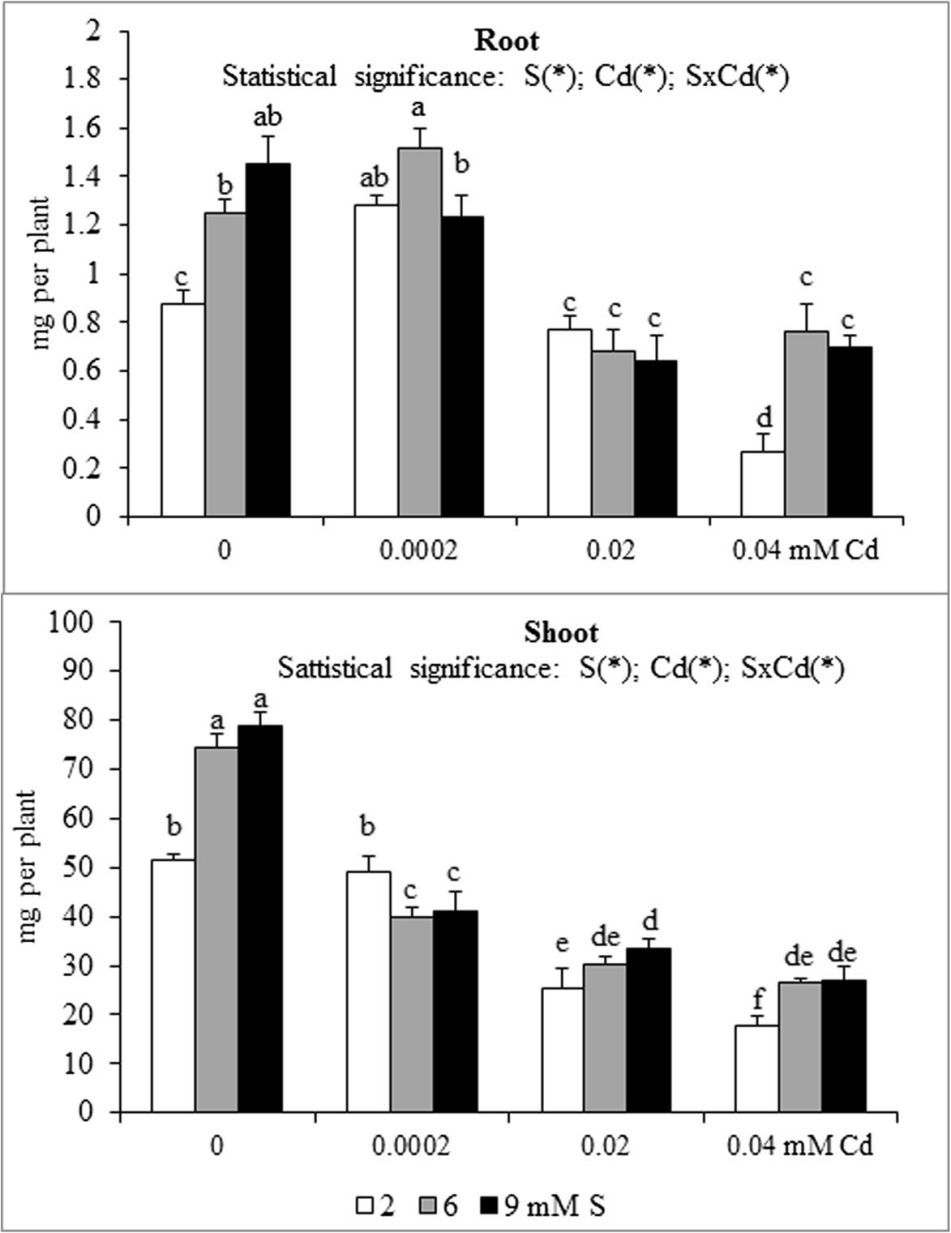
Calcium accumulation in the in the biomass of white mustard ‘Rota’ grown under different sulphur (S) and/or cadmium (Cd) concentrations in the nutrient solution. Note: Explanations are the same as in Fig. 1

**Fig. 5:**
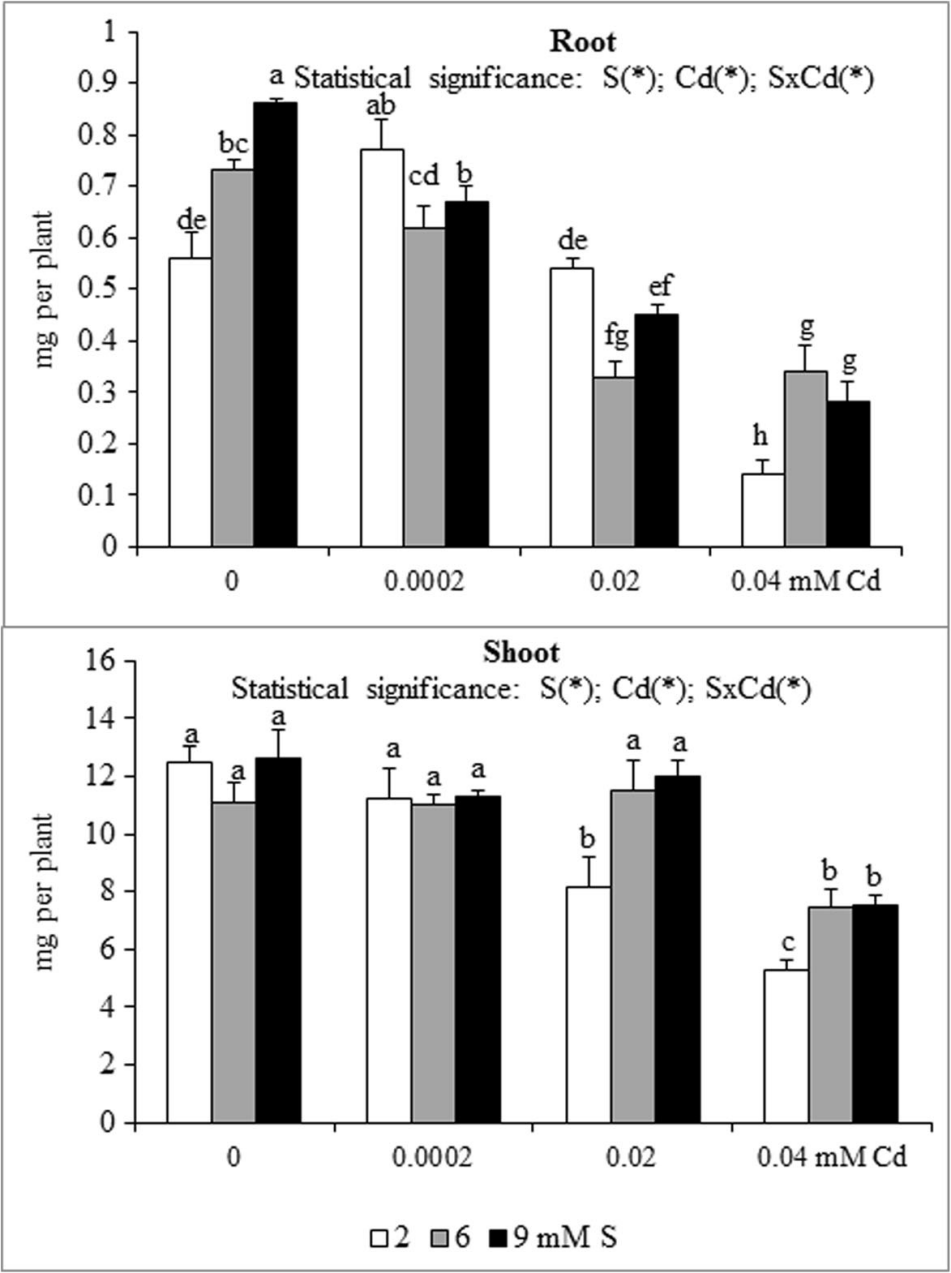
Magnesium accumulation in the in the biomass of white mustard ‘Rota’ grown under different sulphur (S) and/or cadmium (Cd) concentrations in the nutrient solution. Note: Explanations are the same as in Fig. 1

**Fig. 6:**
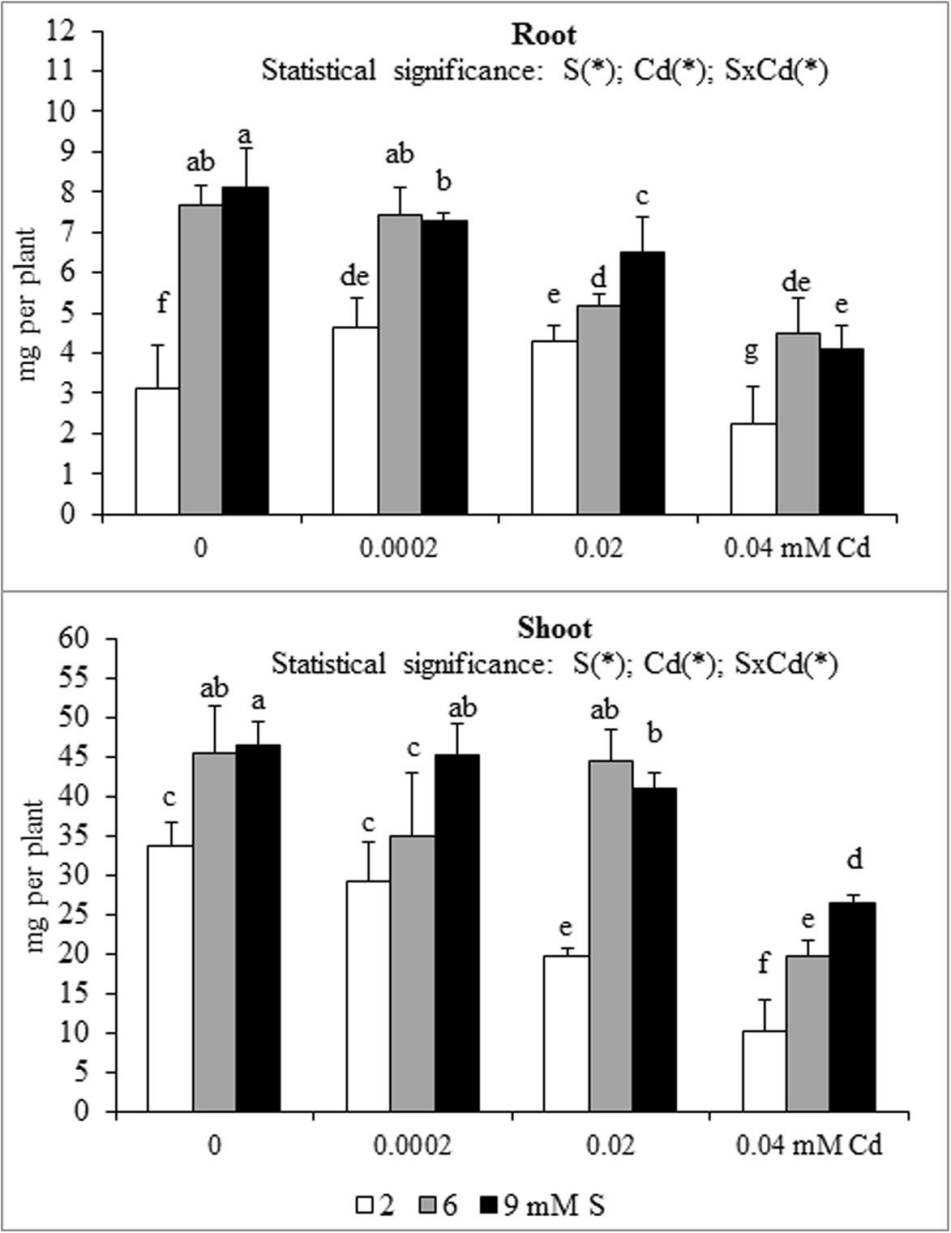
Sulphur accumulation in the in the biomass of white mustard ‘Rota’ grown under different sulphur (S) and/or cadmium (Cd) concentrations in the nutrient solution. Note: Explanations are the same as in Fig. 1

A significant effect of the S and Cd interaction on the root and shoot content and accumulation of macronutrients in mustard was shown (Table 1 and Figs. 1, 2, 3, 4, 5 and 6). An increase in the K and S contents without changed P and Mg levels in the root and shoot biomass of Cd-treated plants supplied with high S doses were found. An exception was significantly elevated root P content under the highest Cd concentration used (0.04 mM). In high S-supplied plants, a decrease in the shoot N content was noted. Moreover, an increase in root N content under the highest Cd concentration was found. It was also established that intensive S nutrition of Cd-stressed mustard resulted in elevated root Ca content under the lowest and the highest Cd concentrations used (0.0002 and 0.04 mM) as well as the decreased content of this macronutrient at the medium Cd treatment (0.02 mM). Under conditions of the lower Cd concentration (0.0002 and 0.02 mM), a substantial drop, whilst at the highest Cd contamination (0.04 mM), no significant changes in the shoot Ca content were revealed (Table 1).

The nutrient accumulation in the mustard biomass results from the changes in the macronutrient content and plants productivity (unpublished data) defined as the amount of dry matter produced by plant during the vegetation. Statistical analysis of main effects showed that in the Cd-exposed plants the N accumulation in mustard roots at 0.0002 or 0.02 mM Cd was elevated, but at 0.04 mM Cd was reduced (Fig. 1). Moreover, the increasing Cd concentrations in the nutrient solution, irrespective of the S level, markedly reduced root P and Mg accumulations (Figs. 2 and 5). It was also shown that the lowest Cd dose (0.0002 mM) raised, but higher doses (0.02 or 0.04 mM) generally decreased the K, Ca and S accumulations in roots (Figs. 3, 4 and 6). Simultaneously, in the shoots of Cd-treated mustard, irrespective of the S level, significantly reduced accumulation of all the macronutrients was found, especially at higher Cd concentrations. The exceptions were the insignificant changes in N, K and Mg accumulations shown at the lowest Cd dose applied (Figs. 1, 2, 3, 4, 5 and 6).

The accumulation of nutrients in Cd-treated plants supplemented with high S levels seems to be a very important issue from the point of view agriculture and horticulture, especially for the members of the mustard family, which accumulate substantial amounts of Cd and are characterized by high requirements for S. Both these elements affect in the different way (Cd inhibits and S promotes) the rate of plant growth and cause various unfavourable (Cd) or beneficial (S) changes in the essential nutrients content and, as consequence, lead to changes in the total accumulation thereof. It should be remembered that the concentration of nutrients, especially S, in the plant biomass is crucial not only for the plant growth and development but also determines plant susceptibility or tolerance to various abiotic and biotic stress factors and, consequently, the quality of crops (Gill et al. 2011; Kumar and Trivedi 2012; Nazar et al. 2012; Upadhyay 2012; Bashir et al. 2015; Singh et al. 2015).

Mustard grown without Cd and intensively fertilized with S showed an increase in the accumulation of all macronutrients in roots. Under these conditions an increased Ca and S contents and decreased K level were noticed in shoots, whereas changes in the N, P and Mg contents were insignificant (Fig. 1, 2, 3, 4, 5 and 6). Moreover, it was found that, regardless of Cd concentration in the nutrient solution, the accumulation of N in mustard intensively supplied with S significantly rose in the roots (Fig. 1). Furthermore, under high S level, irrespective of the Cd concentration, in general, the elevated accumulation of P, K, Ca, Mg and S in the root and shoot was found. The increase in the P, Mg and S accumulations was more pronounced in roots, whilst the rise in K accumulation was much more marked in shoots. The increase in root and shoot Ca accumulation was comparable (Figs. 1, 2, 3, 4, 5 and 6).

A significant effect of the S and Cd interaction on the root and shoot accumulation of macronutrients in mustard was shown (Figs. 2, 3, 4, 5 and 6). It is worth to stress an increase in the accumulation of all macronutrients in shoot biomass of Cd-treated plants supplemented with high S doses (6 or 9 mM), excluding insignificant changes in the shoot N, P and Ca accumulations. Moreover, among others, the considerable increase in the nutrients accumulation recorded in roots of the plants intensively supplied with S and treated with the highest Cd dose should be mentioned. It is worth mentioning that under the presence of the lowest Cd concentration, the fluctuations in the content and the accumulation of the macronutrients were less pronounced. The more marked fluctuations in the content or the accumulation of some macronutrients (N, P, Ca, Mg) in the roots as compared to shoots recorded in the presented studies for the interactions of both experimental factors (S and Cd), may be explained by the fact that the underground organs are directly exposed to the presence of S-SO_4_ and phytotoxic effect of trace metals in the nutrient solution. Thus, the roots are the first line of defence against trace metals.

Thus, the positive effect of high supplementation with S of Cd-treated mustard was in general manifested by increased accumulation of macronutrients in the biomass. This constitutes evidence that S fertilization improves the nutritional environment both in the rhizosphere and plant system, which leads to increased availability of nutrients in the root zone coupled with enhanced metabolic activity (Jat and Mehra 2007; Ganie et al. 2014). The elevated root and shoot accumulation of all macronutrients in Cd-exposed mustard grown under high S doses was mainly caused by the increase in the productivity rather than changes recorded in their content. Only the rise in root S accumulation was related mainly to the elevated content of this macronutrient in these organs.

### Cadmium Accumulation

**Fig. 7:**
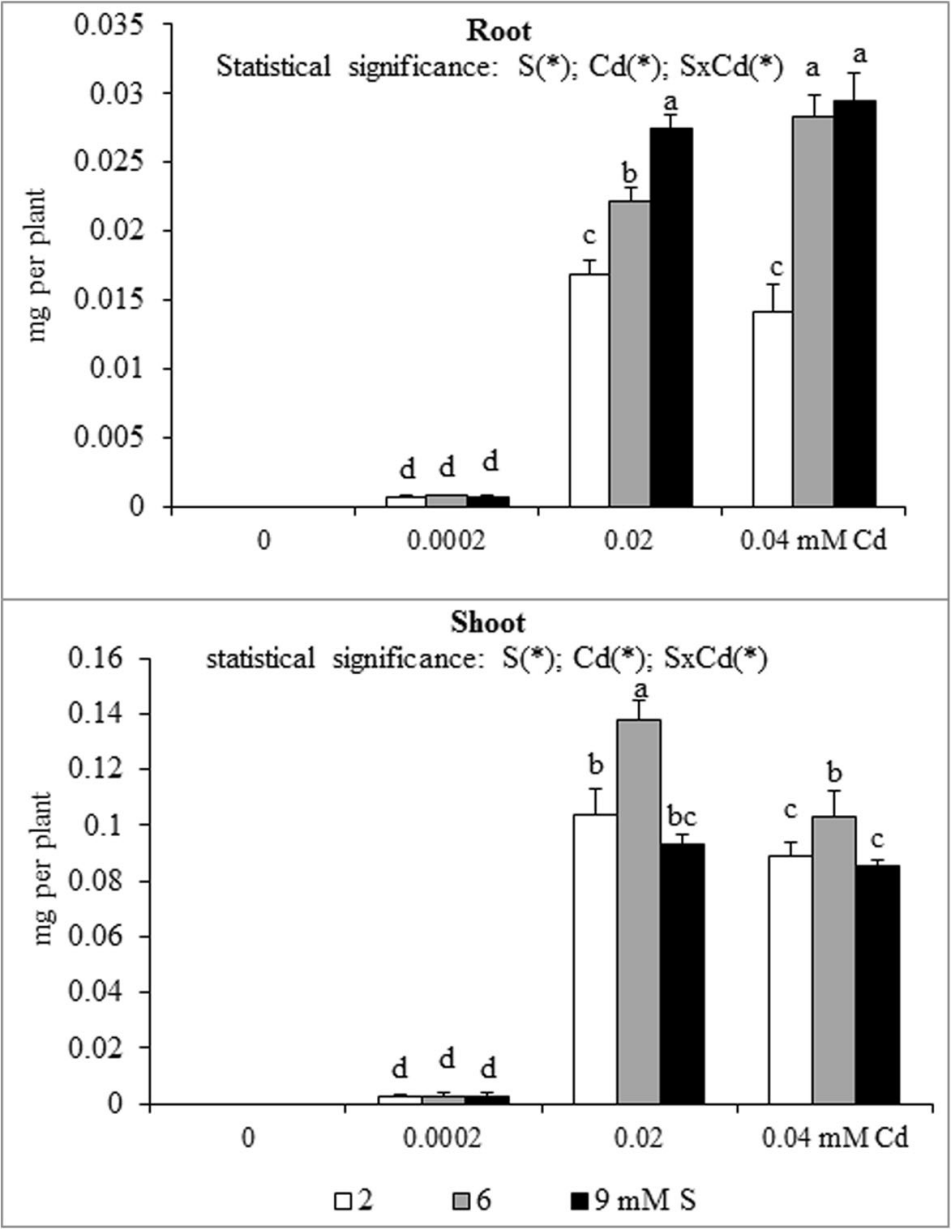
Cadmium accumulation in the in the biomass of white mustard ‘Rota’ grown under different sulphur (S) and/or cadmium (Cd) concentrations in the nutrient solution. Note: Explanations are the same as in Fig. 1. Cadmium concentration in control plants (Cd-0 mM) in basic (S-2) and high-S (S-6 or 9 mM) treated plants was below the detection limit and, hence, accumulations were zero

The results of the present study demonstrated that, irrespective of the S level, Cd accumulation by white mustard substantially rose together with the increasing concentration of this metal in the nutrient solution. This increase was much more pronounced in shoots than in roots and the amount of Cd accumulated in shoots was 3–6 times higher than in roots (Fig. 7). Simultaneously, the Cd content was about 1.5–2.5 times higher in roots than in shoots (data not shown). The increased Cd accumulation was accompanied by a decrease in the dry biomass of shoots and roots (data not shown), wherein the elevated Cd bioaccumulation resulted from the higher Cd content rather than from reduced productivity. A similar pattern of Cd content and accumulation in under- and aboveground parts of different mustard species and cultivars to those found in this study was shown by Salt et al. (1995), Zhu et al. (1999), Jiang et al. (2004), Šimonová et al. (2007) and Gill et al. (2011). Opposite tendencies in the Cd distribution in different parts of mustard plants to that presented in our studies were shown by Singh et al. (2012b), who claim that mustard is unsafe and not suitable for cultivation on Cd-contaminated soils. However, other researchers also recognized mustards, especially Indian mustard, as a high biomass crop species that accumulates substantial amounts of Cd (Salt et al. 1995; Bhadkariya et al. 2014).

It was found that the intensive S nutrition of Cd-stressed mustard significantly raised Cd accumulation in the roots, but only at higher Cd concentrations (0.02 or 0.04 mM). Simultaneously in shoots, depending on the S dose, Cd accumulation increased (at 6 mM S) or was unaffected (at 9 mM S) (Fig. 7). The markedly increased Cd bioaccumulation, especially in shoots of Mexican marigold, with increasing of S levels was revealed also by Feng et al. (2009). This may be related to the fact that an increase in sulphate and elemental S in the culture solution is followed by an increase in active Cd forms and availability of this metal (Skwierawska et al. 2012). The data obtained in our studies shown that enhanced accumulation of Cd in intensive S supplied Cd-stressed mustard was accompanied by the increased Cd content (data in press) as well as by the increased content and S accumulation which may be explained by the elevated synthesis of Cd binding S-ligands involved in the tolerance to Cd phytotoxicity. It worth stressing that in all Cd treatments, the contents of Cd (data in press) in the mustard plant biomass were markedly above the WHO standards, which is 0.2 mg kg^−1^ (Nazir et al. 2015), exceeded the recommended tolerable levels proposed by joint FAO/WHO Expert Committee on Food Additives, which is 0.3 mg kg^−1^ (Satpathy and Reddy 2013), and were higher than the Cd range proposed by the EU Commission Decree, i.e. 0.05–0.2 mg kg^−1^ (Dymkowska-Malesa et al. 2007). Other researchers also mention lower acceptable limits in plants than those recorded in the presented studies (Singh et al. 2012b; Shah et al. 2013).

## Conclusions

To conclude, S-Cd interactions play a crucial role in white mustard ‘Rota’ macronutrient status. Our research demonstrated that Cd exposure (0.0002–0.04 mM) results in various unfavourable changes in the macronutrient content and accumulation as well as raises Cd bioaccumulation in the roots and shoots of mustard. High S supplementation (6 or 9 mM) appears to some extent to have a beneficial effect on the macronutrient status of Cd-stressed mustard. In general, it substantially increased the K and S contents in roots and shoots. Simultaneously, the Mg content generally was stable under different Cd and S treatments. In turn, shoot Ca content decreased at 6 mM S, whilst root Ca content rose at 9 mM S. Furthermore, intensive S nutrition of Cd-stressed mustard increased root N content and accumulation at the highest Cd treatment, whilst the N content dropped in shoots. The bioaccumulation of remaining macronutrients, i.e. P, K, Ca, Mg and S, in general was substantially elevated. Also, Cd accumulation was enhanced, especially in roots of plants exposed to the highest Cd concentration. We believe that our study are important for farmers and provide some new valuable information on the strategies developed by plants to cope with Cd.
